# Larger Numbers of Glial and Neuronal Cells in the Periaqueductal Gray Matter of μ-Opioid Receptor Knockout Mice

**DOI:** 10.3389/fpsyt.2018.00441

**Published:** 2018-09-19

**Authors:** Kazumasu Sasaki, Frank Scott Hall, George R. Uhl, Ichiro Sora

**Affiliations:** ^1^Department of Preclinical Evaluation, Institute of Development, Aging and Cancer, Tohoku University, Sendai, Japan; ^2^Department of Pharmacology and Experimental Therapeutics, College of Pharmacy and Pharmaceutical Sciences, The University of Toledo, Toledo, OH, United States; ^3^Raymond G Murphy VA Medical Center, Albuquerque, NM, United States; ^4^Department of Psychiatry, Kobe University Graduate School of Medicine, Kobe, Japan

**Keywords:** μ opioid, μ opioid receptor knockout (MOP-KO), periaqueductal gray matter (PAG), microglia, astrocytes, neuron, immunohistochemistry

## Abstract

**Background:** μ-opioid receptor knockout (MOP-KO) mice display baseline hyperalgesia. We have recently identified changes in tissue volume in the periaqueductal gray matter (PAG) using magnetic resonance imaging voxel-based morphometry. Changes in the structure and connectivity of this region might account for some behavior phenotypes in MOP-KO mice, including hyperalgesia.

**Methods:** Adult male MOP-KO and wild-type (WT) mice were studied. Immunohistochemistry was performed to detect microglia, astrocytes, and neurons in the PAG using specific markers: ionized calcium-binding adaptor molecule 1 (Iba-1) for microglia, glial fibrillary acidic protein (GFAP) for astrocytes, and the neuronal nuclei antigen (NeuN; product of the Rbfox3 gene) for neurons, respectively. Cell counting was performed in the four parallel longitudinal columns of the PAG (dorsomedial, dorsolateral, lateral, and ventrolateral) at three different locations from bregma (−3.5, −4.0, and −4.5 mm).

**Results:** The quantitative analysis showed larger numbers of well-distributed Iba1-IR cells (microglia), NeuN-IR cells (neurons), and GFAP-IR areas (astrocytes) at all the anatomically distinct regions examined, namely, the dorsomedial (DM) PAG, dorsolateral (DL) PAG, lateral (L) PAG, and ventrolateral (VL) PAG, in MOP-KO mice than in control mice.

**Conclusions:** The cellular changes in the PAG identified in this paper may underlie aspects of the behavioral alterations produced by MOP receptor deletion, and suggest that alterations in the cellular structure of the PAG may contribute to hyperalgesic states.

## Introduction

Studies in knockout (KO) mice have demonstrated that μ-opioid (MOP) receptors play crucial roles in several physiological functions, including nociception, stress responses, tolerance, reward learning, and immune function (1–5). Although these effects of MOP deletion have been generally thought to result simply from elimination of MOP signaling (6), as would be expected from elimination of opiate reinforcement in MOP-KO mice (7, 8), effects on drug reinforcement extend to abused drugs acting through diverse mechanisms [(9–11), see summary in Hall et al. (12)]. This may still just indicate a role for MOP in drug reinforcement generally, just as baseline hyperalgesia may indicate a role of MOP in basal nociception (1, 8). However, there certainly is evidence for neuroadaptations to the elimination of MOP. Most recently, we have found brain volume differences in the periaqueductal gray matter (PAG), olfactory bulb, arcuate nucleus, and several cerebellar regions using magnetic resonance imaging voxel-based morphometry (13).

Several factors are likely to be involved in the brain volume abnormalities in the PAG caused by the deletion of MOP receptors. The brains of several strains of mutant mice exhibit structural changes, that correlate with behavioral consequences of the genetic modifications (14). Since glial cells take up a large portion of neural tissue, it is likely that glial changes may account for some of these differences in tissue volume (although that does not exclude changes in the volume of the neuropil as well, which would be likely to drive changes in glial numbers or volume). Indeed, glial changes are found in altered pain states in the spinal cord, as well as marked changes in specific brain regions (15–17). Changes in some regions may involve a role of opioids in brain development, but others may involve adult plasticity. Hippocampal neurogenesis is affected by MOP deletion (18). Although opioid systems modulate neural stem cell progenitor differentiation and influence aspects of neural development, it is important to note that opioid agonists also affect neurogenesis in adult animals (19, 20), indicating that these effects are not necessarily developmental in nature. Current evidence support genetic factors affect brain functional connectivity and organization (21). MOP receptor gene alters the widespread brain functional connectome and remodels the reward/aversion circuit (22). Such connectivity remodeling may account for brain morphology alterations.

Thus, it is likely that alterations in MOP signaling may have broader effects on developmental and adult neuroplasticity, in addition to simply altering MOP activity. This might be evidenced as altered brain morphology and connectivity. In our previous study, finding increased size of the PAG in MOP-KO mice, histological analysis did not reveal apparent cellular pathological changes, based on conventional hematoxylin and eosin/Klüver-Barrera staining, although there were increased neural cell numbers (13). Consequently, the aim of the present study was to investigate the contribution of different neural cell types to the volume and cell number differences in the PAG resulting from genetic elimination of MOP receptors.

## Materials and methods

### Animals

All animals were treated in compliance with the “Principles of Laboratory Animal Care” (National Society for Medical Research) and the “Guide for the Care and Use of Laboratory Animals” (National Academies of Sciences). The Animal Care and Use Committee of the Tohoku University Graduate School of Medicine approved this study.

Congenic homozygous male MOP-KO (N = 7) and wild-type (WT, N = 7) mice that had been backcrossed for at least 20 generations to C57BL/6J mice were used (1). All mice were housed at the Institute for Animal Experimentation, Tohoku University Graduate School of Medicine, in a colony maintained at an ambient temperature of 22 ± 2°C, on a 12 h light:12 h dark cycle (lights on: 08:00–20:00) with food and water available *ad libitum*. Four to six mice were housed per cage. All mice were 12 weeks old at the time of sacrifice for immunohistochemical analysis.

### Immunohistochemistry

Each mouse was anesthetized by intraperitoneal administration of a combination of medetomidine (0.3 mg/kg, Medetomin; Meiji Seika Pharma, Co., Ltd., Tokyo, Japan) and butorphanol (2 mg/kg, Betrorphal; Meiji Seika Pharma, Co., Ltd., Tokyo, Japan). Local anesthesia, with 2% lidocaine (diluted to 0.5%, 3 mg/kg), was performed at the incision site. Animals were perfused transcardially with cold 0.1 M phosphate-buffered saline (PBS, pH 7.4) followed by 4% paraformaldehyde in 0.1 M PBS for 30 min, at rate of 7 mL/min. After perfusion, the brains were removed and post-fixed in 4% paraformaldehyde in 0.1 M PBS. After post-fixation, tissues were embedded in paraffin using a specialized automated tissue processing system (Tissue-Tek, Sakura Finetek Japan Co., Ltd., Tokyo, Japan) at 58°C; 5-μm coronal sections were cut from the three anatomically distinct regions of the PAG (bregma: −3.5, −4.0, and −4.5 mm) for each of the brains from MOP-KO and WT mice (23). For each of the three regions, 5 serial sections (total number of sections: 15 per mouse) were collected.

Each formalin-fixed and paraffin wax-embedded tissue section was cleaned in xylene and rehydrated with decreasing concentrations of ethanol. For studying microglia, each section was subjected to a standard antigen retrieval procedure consisting of 5 min autoclaving at 120°C in antigen retrieval buffer, using pretreatment regent (Deparaffinization/Antigen Retrieval Solution, pH 9; Nichirei Bioscience, Tokyo, Japan), for ionized calcium-binding adapter molecule 1 (Iba1). The sections were cooled at 4°C for 30–45 min and incubated with the primary antibody (anti-Iba1 antibody, goat polyclonal, 1:2,000; Abcam, Tokyo, Japan) overnight at 4°C. The next day, the sections were washed three times with 0.01 M PBS (10 min per wash), and endogenous enzyme activity was blocked using 1% H_2_O_2_ for 20 min. Each section was stained using the indirect immunoperoxidase method (Histofine Simple Stain Max PO (G); Nichirei Bioscience), and a chromogen complex, 3,3′-diaminobenzidine tetrachloride (Simple Stain DAB Solution; Nichirei Bioscience) was used to visualize the targeted antigens; the sections were then counterstained with hematoxylin (Chroma, Köngen, Germany).

To label astrocytes, a similar protocol was used for polyclonal rabbit anti-glial fibrillary acidic protein (GFAP) (1:2,000; Dako, Tokyo, Japan). Immunoreactivity was examined using the indirect immunoperoxidase method (Histofine Simple Stain Max PO (R); Nichirei Bioscience).

In order to detect neuronal nuclei (NeuN) expression in neurons, the antigen was retrieved by heating the samples in a microwave for 15 min at 100°C in 0.01 mol/L citrate buffer (pH 6.0). After the slides were washed, they were incubated with Blocking Reagent A (Nichirei Bioscience) for 1 h at room temperature. The sections were incubated with primary antibody (Anti-NeuN antibody, mouse monoclonal, 1:1,000; Millipore, CA, USA) overnight at 4°C. The next day, the sections were washed three times with 0.01 M PBS (10 min per wash) and endogenous enzyme activity was blocked using 1% H_2_O_2_ for 20 min. Each section was stained using the indirect immunoperoxidase method (Histofine Simple Stain Max PO (M); Nichirei Bioscience), and a chromogen complex, 3,3′-diaminobenzidine tetrachloride (Simple Stain DAB Solution, Nichirei Bioscience) was used to visualize the targeted antigens; the sections were then counterstained with hematoxylin, followed by incubation with Blocking Reagent B (Nichirei Bioscience).

The immunoreactivity of each antibody (GFAP, Iba1, and NeuN) for paraffin-embedded sections was confirmed using the procedures recommended in each product's data sheet.

### Image analysis

The number of immunoreactive (IR) nuclei (for Iba1-IR and NeuN-IR, that show distinct cells) and the immunoreactive area (for GFAP-IR, that shows a more diffuse staining) were quantified using a light microscope equipped with a computer-based automated cell counting system (BZ-9000, KEYENCE, Tokyo, Japan) at the four columns in the PAG (dorsomedial: DM, dorsolateral: DL, lateral: L, and ventrolateral: VL) and three different locations from the bregma (−3.5, −4.0, and −4.5 mm) in 300 × 300 μm^2^ fields, following standard mouse brain coordinates (23). While viewing the automated cell counting system monitor, upper and lower thresholds of immunostaining gray level were set such that only Iba1-IR, GFAP-IR and NeuN-IR was accurately discriminated from the background in outlined PAG area. The boundaries of each PAG column were defined based on previously published anatomical criteria (24, 25).

### Statistical analysis

A P-value of 0.05 was considered statistically significant. The Mann-Whitney U-test with *post hoc* Bonferroni-Dunn corrected means comparisons were used to evaluate individual group differences. Statistical analyses were performed using IBM SPSS Statistics 2.4 (IBM, Chicago, IL, USA), GraphPad Prism Version 7.0 (GraphPad Software, Inc., La Jolla, CA, USA), and SigmaPlot Version 13.0 (Systat Software, Inc., CA, USA). Data is presented as median (interquartile range), unless mentioned otherwise.

## Results

**Figure 1:**
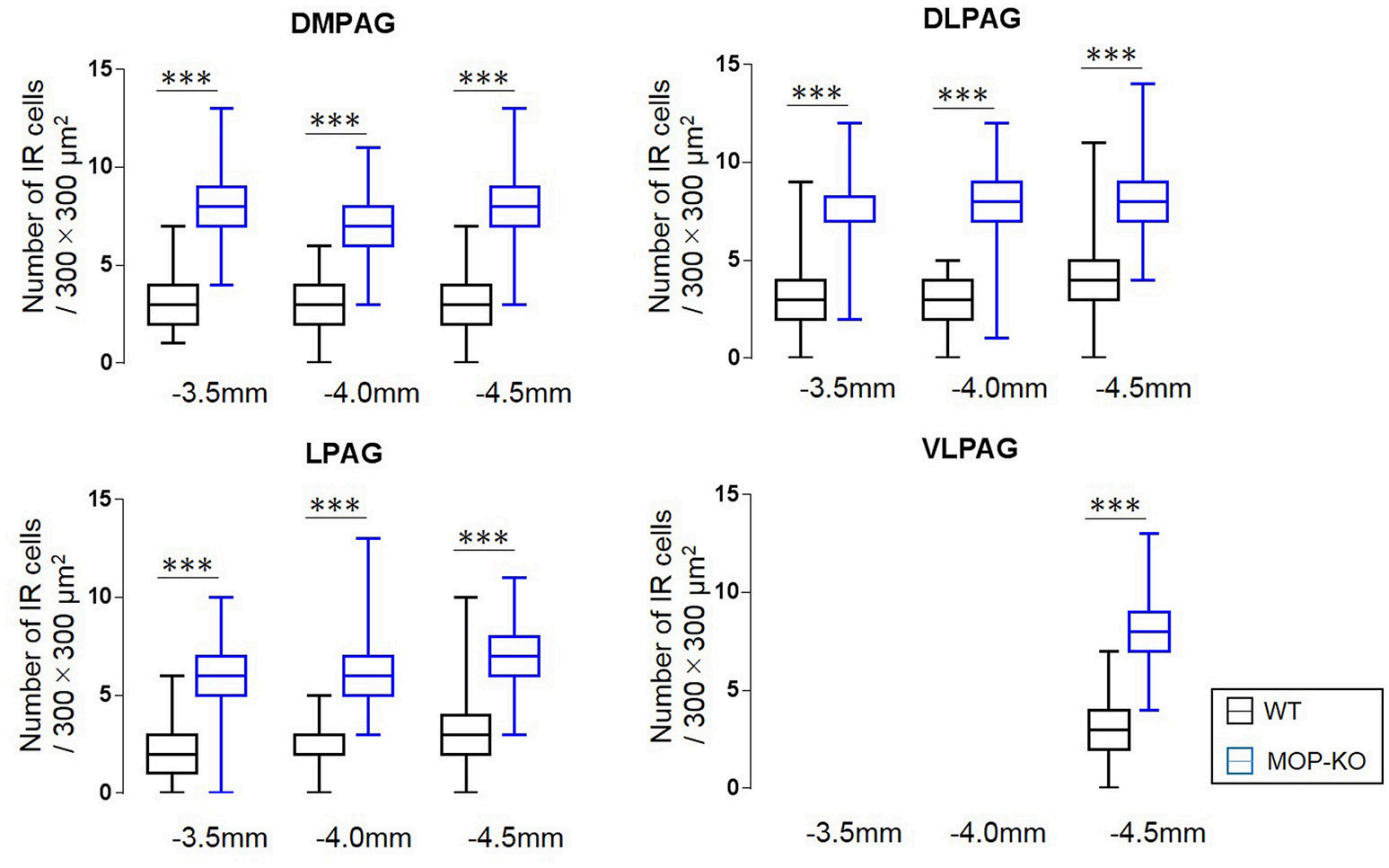
Median values and interquartile ranges for the number of Iba1-IR cells in the examined fields (300 × 300 μm^2^) in the DMPAG (−3.5, −4.0, and −4.5 mm from bregma), DLPAG (−3.5, −4.0, and −4.5 mm from bregma), LPAG (−3.5, −4.0, and −4.5 mm from bregma), and VLPAG (−4.5 mm from bregma). Fifteen slides per brain tissue block (N = 7) were designated for image analysis. The asterisk indicates significant differences (MOP-KO vs. WT mice). Iba1, ionized calcium-binding adapter molecule 1; IR, immunoreactive; DMPAG, dorsomedial periaqueductal gray matter (PAG); DLPAG, dorsolateral PAG; LPAG, lateral PAG; VLPAG, ventrolateral PAG. ***P < 0.001.

The quantitative analysis showed larger numbers of well-distributed microglia (Iba1-IR), neurons (NeuN-IR) and astrocytes (GFAP-IR) at all the anatomically distinct regions examined, namely, the DMPAG, DLPAG, LPAG, and VLPAG, in MOP-KO mice than in control mice. The analysis showed that MOP-KO mice displayed greater numbers of Iba1-IR cells at −3.5 mm from bregma (WT: 3 [2–4] vs. MOP-KO: 8 [7–9], P < 0.001, n = 105), −4.0 mm from bregma (WT: 3 [2–4] vs. MOP-KO: 7 [6–8], P < 0.001, n = 105), and −4.5 mm from bregma (WT: 3 [2–4 vs. MOP-KO: 8 [7–9], P < 0.001, n = 105) in the DMPAG; −3.5 mm from bregma (WT: 3 [2–4] vs. MOP-KO: 7 [6–8], P < 0.001, n = 105), −4.0 mm from bregma (WT: 3 [2–4] vs. MOP-KO: 8 [7–9], P < 0.001, n = 105), and −4.5 mm from bregma (WT: 4 [3–5] vs. MOP-KO: 8 [7–9], P < 0.001, n = 105) in the DLPAG; −3.5 mm from bregma (WT: 2 [1–3] vs. MOP-KO: 6 [5–7], P < 0.001, n = 105), −4.0 mm from bregma (WT: 2 [1–3] vs. MOP-KO: 6 [5–7], P < 0.001, n = 105), −4.5 mm from bregma (WT: 3 [2–4] vs. MOP-KO: 7 [6–8], P < 0.001, n = 105) in the LPAG; and −4.5 mm from bregma (WT: 3 [2–4] vs. MOP-KO: 8 [7–9], P < 0.001, n = 105) in the VLPAG. These data are illustrated in Figure 1 (also see the representative photomicrographs in Figure 4).

**Figure 2:**
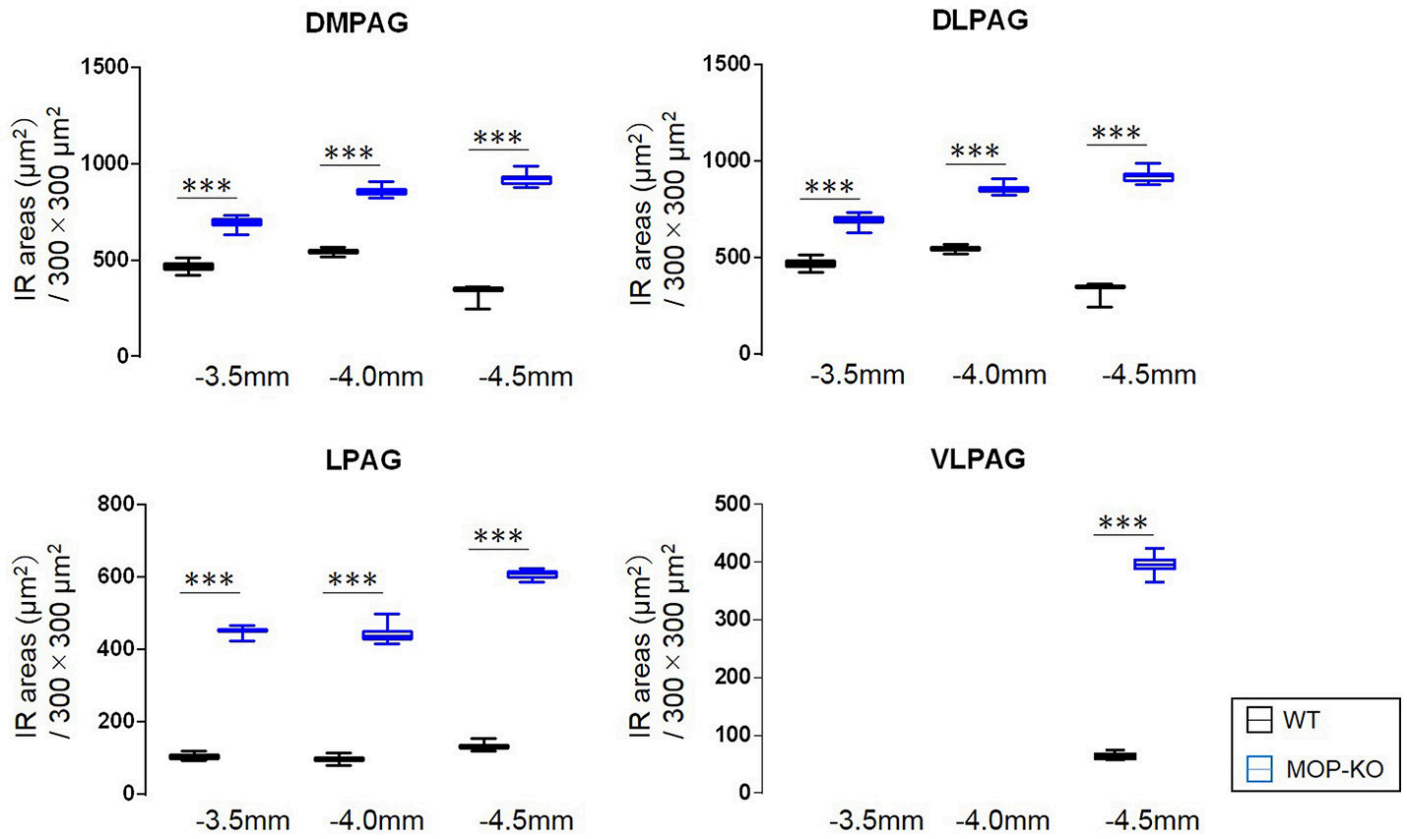
Median values and interquartile ranges of the GFAP-IR areas (μm^2^) in the examined fields (300 × 300 μm^2^) in the DMPAG (−3.5, −4.0, and −4.5 mm from bregma), DLPAG (−3.5, −4.0, and −4.5 mm from bregma), LPAG (−3.5, −4.0, and −4.5 from bregma), and VLPAG (−4.5 mm from bregma). Fifteen slides per brain tissue block (N = 7) were designated for image analysis. The asterisk indicates significant differences (MOP-KO vs. WT). GFAP, glial fibrillary acidic protein; DMPAG, dorsomedial periaqueductal gray matter (PAG); DLPAG, dorsolateral PAG; LPAG, lateral PAG; VLPAG, ventrolateral PAG. ***P < 0.001.

MOP-KO mice displayed larger GFAP-IR area (μm^2^) at −3.5 mm from bregma (WT: 471.0 [455.8–486.0] vs. MOP-KO: 699.0 [685.0–711.3], P < 0.001, n = 105), −4.0 mm from bregma (WT: 548.0 [538.8–554.3] vs. MOP-KO: 854.5 [843.8–866.3], P < 0.001, n = 105), and −4.5 mm from bregma (WT: 351.0 [344.0–357.3] vs. MOP-KO: 923.0 [899.0–934.0], P < 0.001, n = 105) in the DMPAG; −3.5 mm from bregma (WT: 60.0 [58–62.0] vs. MOP-KO: 158.0 [153.0–164.3], P < 0.001, n = 105), −4.0 mm from bregma (WT: 63.0 [59.8–66.0] vs. MOP-KO: 248.5 [243.0–251.0], P < 0.001, n = 105), and −4.5 mm from bregma (WT: 51.0 [49.0–53.0] vs. MOP-KO: 350.5 [349.0–354.3], P < 0.001, n = 105) in the DLPAG; −3.5 mm from bregma (WT: 102.0 [99.0–108.3] vs. MOP-KO: 451.0 [450.0–455.0], P < 0.001, n = 105), −4.0 mm from bregma (WT: 99.0 [94.0–102.0] vs. MOP-KO: 437.0 [429.8–450.0], P < 0.001, n = 105), and −4.5 mm from bregma (WT: 131.0 [128.0–137.0] vs. MOP-KO: 609.5 [600.8–617.0], P < 0.001, n = 105) in the LPAG; and −4.5 mm from bregma (WT: 62.5 [59.8–68.0] vs. MOP-KO: 395.0 [388.8–403.0], P < 0.001, n = 105) in the VLPAG. These data are illustrated in Figure 2 (also see the representative photomicrographs in Figure 4).

**Figure 3:**
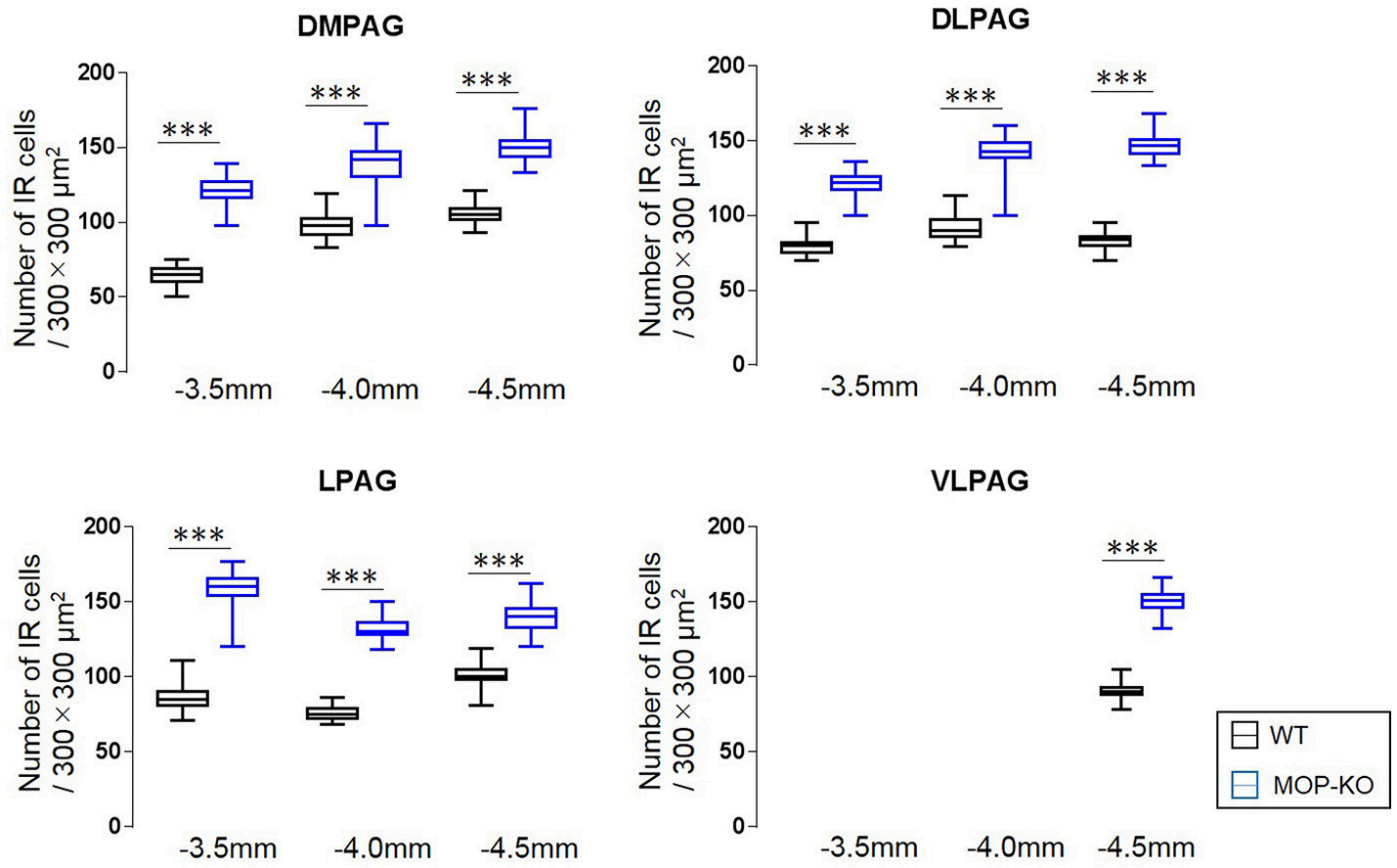
Median values and interquartile ranges for the number of NeuN-IR cells in the examined fields (300 × 300 μm^2^) in the DMPAG (−3.5, −4.0, and −4.5 mm from bregma), DLPAG (−3.5 mm, −4.0 mm, and −4.5 mm from bregma), LPAG (−3.5, −4.0, and −4.5 mm from bregma), and VLPAG (−4.5 mm from bregma). Fifteen slides per brain tissue block (N = 7) were designated for image analysis. The asterisk indicates significant differences (MOP-KO vs. WT). NeuN, neuronal nuclei; DMPAG, dorsomedial periaqueductal gray matter (PAG); DLPAG, dorsolateral PAG; LPAG, lateral PAG; VLPAG, ventrolateral PAG. ***P < 0.001.

**Figure 4:**
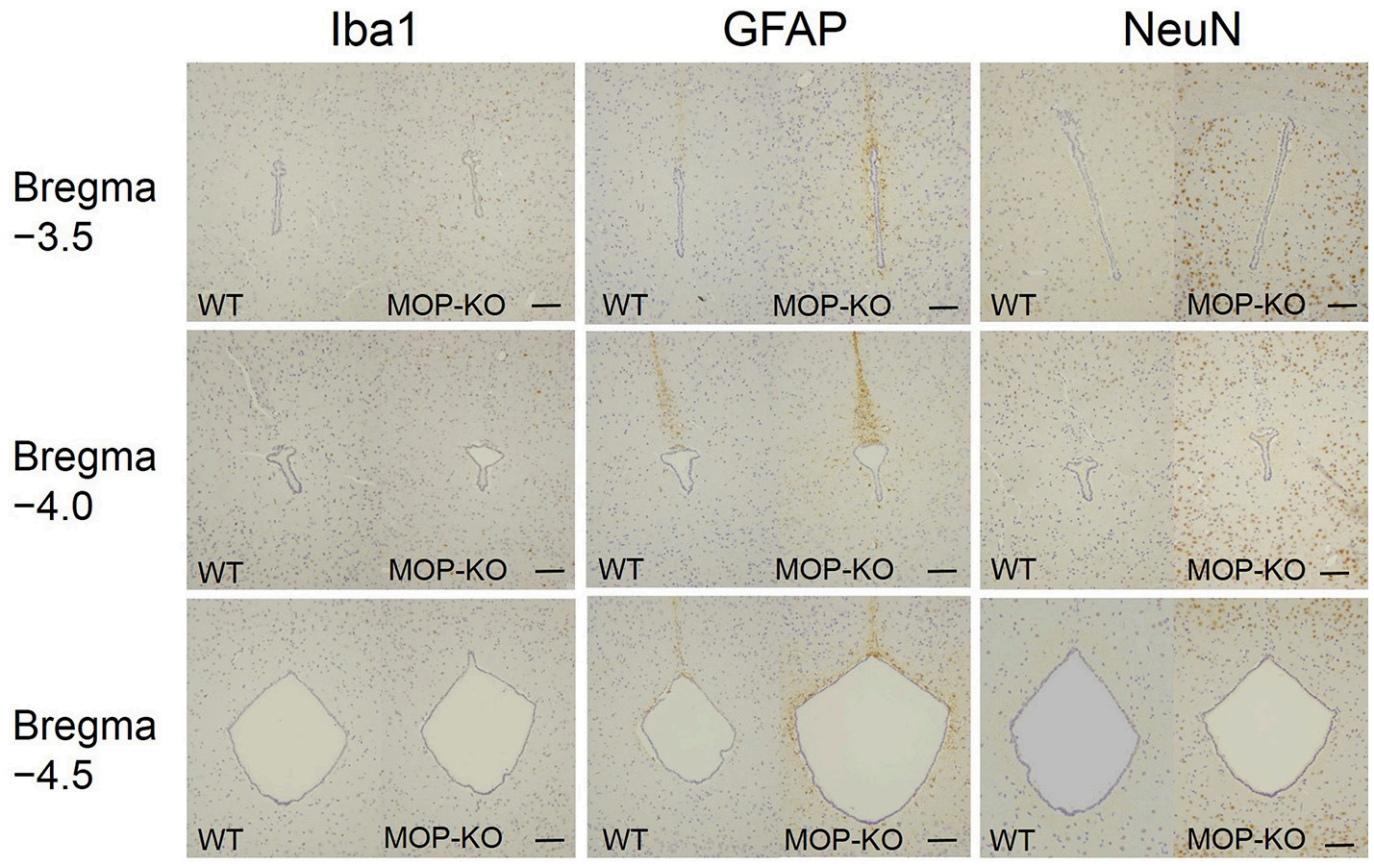
Representative photomicrographs of Iba1-IR cells, GFAP-IR areas, and NeuN-IR cells in the PAG. Iba1, ionized calcium-binding adapter molecule 1; IR, immunoreactive; GFAP, glial fibrillary acidic protein; NeuN, neuronal nuclei; PAG, periaqueductal gray matter. Scale bars indicate 100 μm.

The analysis showed that MOP-KO mice had larger numbers of NeuN-IR cells at −3.5 mm from bregma (WT: 65.0 [60.5–69.0] vs. MOP-KO: 121.0 [116.5–127.0], P < 0.001, n = 105), −4.0 mm from bregma (WT: 98.0 [91.5–102.5] vs. MOP-KO: 142.0 [130.5–147.0], P < 0.001, n = 105), and −4.5 mm from bregma (WT: 105.0 [102.0–109.0] vs. MOP-KO: 150.0 [144.0–154.5], P < 0.001, n = 105) in the DMPAG; −3.5 mm from bregma (WT: 80.0 [75.0–82.0] vs. MOP-KO: 122.0 [117.0–126.0], P < 0.001, n = 105), −4.0 mm from bregma (WT: 90.0 [86.0–97.0] vs. MOP-KO: 143.0 [138.5–149.0], P < 0.001, n = 105), and −4.5 mm from bregma (WT: 84.0 [80.0–86.0] vs. MOP-KO: 147.0 [141.5–151.0], P < 0.001, n = 105) in the DLPAG; −3.5 mm from bregma (WT: 85.0 [81.0–90.0] vs. MOP-KO: 160.0 [154.0–165.0], P < 0.001, n = 105), −4.0 mm from bregma (WT: 75.0 [72.0–79.0] vs. MOP-KO: 130.0 [128.0–136.0], P < 0.001, n = 105), and −4.5 mm from bregma (WT: 100.0 [98.0– 105.0] vs. MOP-KO: 140 [133.0–145.5], P < 0.001, n = 105) in the LPAG; and −4.5 mm (WT: 90.0 [88.0–93.0] vs. MOP-KO: 151.0 [146.0–155.0], P < 0.001, n = 105) in the VLPAG. These data are illustrated in Figure 3 (also see the representative photomicrographs in Figure 4).

## Discussion

The aim of this study was to determine the contribution of changes in the numbers of different neural cell types (or area for astrocytes) to the volume changes in the PAG of MOP-KO mice. Immunohistochemical analysis revealed that enlarged brain size was accompanied by an increase in the number of microglia and neurons, and area of astrocyte immunoreactivity, in all of the anatomically distinct regions of the PAG that were examined (these included three different locations from the bregma [−3.5, −4.0, and −4.5 mm] and four different columns in the PAG [dorsomedial, dorsolateral, lateral, and ventrolateral].

Regulation of neurodevelopment by the endogenous opioid system is an important concept for the interpretation of our findings. Firstly, it is of interest to note that opioid receptor blockade increases DNA synthesis in germinal neural cells (26). Opioid antagonists exert a marked, stereospecific influence on the growth of neural tissues, depending on the duration of opioid receptor blockade (27). Continuous daily blockade of opioid receptors increases the number of cells in the cerebellum, whereas intermittent opioid receptor blockade decreases the number of these cells (26). Quantitative analysis has demonstrated that all cerebellar cell types, including granule cells, Purkinje cells, and glial cells, contribute to this cerebellar plasticity (28). Given these facts, our findings are in line with the cerebellar structural changes induced by continuous daily blockade of opioid receptor using naltrexone in postnatal rats (27).

However, the specific mechanisms by which MOP receptors regulate neural development in specific brain areas is yet to be determined, and likely to involve more than just MOP receptor density. Distribution and levels of MOP receptors in the brain do not predict those areas in which increased cell numbers are observed, such as the cerebellum and PAG, since neither of those areas contain high levels of MOP receptors (29), and many regions with high levels of MOP expression do not seem to be affected.

Our results indicate that structural abnormalities in the PAG may not relate to the anatomical locations and cellular organization of PAG circuits because increased cell numbers were observed in all cell types, in all PAG columns—there is just more of everything. Each anatomical subdivision of the PAG does have a role in distinct physiological functions that include the control and expression of pain, analgesia, fear, and anxiety (30, 31). Briefly, dorsolateral PAG stimulation evokes active coping strategies, such as fight/flight behaviors, non-opioid-mediated analgesia, hypertension, and tachycardia. The lateral PAG appears to coordinate non-opioid analgesia, active defensive behaviors, and exerts a hypertensive effect. Ventrolateral stimulation, on the other hand, evokes passive defensive behaviors, such as quiescence, opioid-mediated analgesia, hypotension, and bradycardia (25, 31, 32). Our findings do not necessarily indicate a large shift in these behavioral circuits mediated by PAG since all areas were affected, although these areas were not entirely equally affected. It will require more work to determine if these small differences affect the activity of individual PAG subregions and the behavior circuits that they influence.

A number of studies have demonstrated the importance of several brain sites in modulating nociception and/or mediating analgesic effects, including the PAG (33, 34), thalamus (35), hypothalamus, and amygdala (34). It is well-known that the endogenous descending pain modulatory circuit originates in the PAG and includes neurons in the rostral ventromedial medulla and spinal cord dorsal horn (36). Evidence indicates that glial plasticity in specific brain areas affect pain states, beyond the changes in numbers of glia occurring in the spinal cord and the peripheral nerves (15–17). Marked structural glial modifications may also occur in the PAG of MOP-KO mice that have baseline hyperalgesia, but the present study demonstrates that increased numbers of neuronal cells also contribute to volume abnormalities in that brain region. Neuronal changes in specific brain areas associated with chronic pain states, including hyperalgesia and allodynia, remain to be fully elucidated, so the extent to which these changes are specifically involved in hyperalgesia remains to be seen.

Our study has some limitations. Firstly, the examination was performed at 12 weeks of age, and our findings require corroboration from investigations at earlier stages of life. Secondly, increased regional gray matter volume in MOP-KO mice was observed in other brain regions, such as the hypothalamus and olfactory bulb (13). Further investigation of these brain regions is warranted to determine whether changes in all neural cell types are also detected in these regions as well. Thirdly, we did not examine baseline cytokine levels, or other potential mediators beyond MOP in MOP-KO mice that show baseline hyperalgesia. Specific cytokines may influence the numbers of glial cells observed in the PAG due to the deletion of MOP receptors. Finally, further study is warranted to clarify the sex-bias in each genotype since we did not analyze the data by sex.

In conclusion, the present study shows that increased numbers of microglia, and neurons, and greater astrocytic area, in the PAG in MOP-KO mice might result from developmental roles of by the endogenous opioid system. Further investigations based on the present findings are necessary to elucidate whether structural changes are observed earlier in life, and if so, why these changes are induced in specific brain areas, and what phenotypic outcomes are mediated by these cellular changes.

## Author contributions

KS study design, immunohistochemistry, data interpretation, statistical analysis, and preparation of the manuscript. FH, GU, and IS data interpretation and preparation of the manuscript. All authors have read and approved the manuscript.

### Conflict of interest statement

The authors declare that the research was conducted in the absence of any commercial or financial relationships that could be construed as a potential conflict of interest.
